# Pharmacokinetics and safety of carfilzomib in patients with relapsed multiple myeloma and end-stage renal disease (ESRD): an open-label, single-arm, phase I study

**DOI:** 10.1007/s00280-017-3287-8

**Published:** 2017-04-19

**Authors:** Hang Quach, Darrell White, Andrew Spencer, P. Joy Ho, Divaya Bhutani, Mike White, Sandeep Inamdar, Chris Morris, Ying Ou, Martin Gyger

**Affiliations:** 1 St. Vincent’s Hospital Melbourne, 41 Victoria Parade, Fitzroy, VIC 3065 Australia; 2 University of Melbourne, Parkville, VIC 3010 Australia; 3 Dalhousie University and Queen Elizabeth II Health Sciences Centre, 1276 South Park St, Halifax, NS B3H 2Y9 Canada; 4 Malignant Haematology and Stem Cell Transplantation Service, The Alfred Hospital, 55 Commercial Rd, Melbourne, VIC 3004 Australia; 5 Institute of Haematology, Royal Prince Alfred Hospital, Missenden Rd, Camperdown, NSW 2050 Australia; 6 Bosch Institute, University of Sydney, Sydney, NSW 2006 Australia; 7 Barbara Ann Karmanos Cancer Institute, 4100 John R St, Detroit, MI 48201 USA; 8 Amgen Inc., 1120 Veteran Blvd, South San Francisco, CA 94080 USA; 9 Jewish General Hospital, 3755 Chemin de la Côte-Sainte-Catherine, Montreal, QC H3T 1E2 Canada

**Keywords:** Multiple myeloma, Carfilzomib, Pharmacokinetics, Renal impairment, End stage renal disease

## Abstract

**Purpose:**

The pharmacokinetics (PK) of carfilzomib have been previously studied in multiple myeloma patients with varying degrees of renal impairment (normal, mild, moderate, severe, and end-stage renal disease [ESRD]) at doses of 15 and 20 mg/m^2^. This study evaluated carfilzomib PK at higher doses of 27 and 56 mg/m^2^ in normal renal function and ESRD patients.

**Methods:**

Patients received carfilzomib on two consecutive days/week for 3 weeks every 28-day cycle: 20 mg/m^2^ (cycle 1 day 1–2), escalated to 27 mg/m^2^ on cycle 1 day 8; if tolerated, 56 mg/m^2^ starting cycle 2 day 1. The primary objective was PK assessment with safety/tolerability and response rate as secondary and exploratory objectives, respectively.

**Results:**

26 patients were enrolled (15 normal, 11 ESRD). There was a trend toward higher area under the concentration time curve (AUC) and maximum concentration in ESRD versus normal renal function patients; however, high interpatient PK variability was discerned. Relative to patients with normal renal function, ESRD patients showed 33% higher AUC. Overall response rate was 43% for the normal renal function and 60% for the ESRD groups. Safety findings were generally similar between the two groups and consistent with the known safety profile of carfilzomib in multiple myeloma patients.

**Conclusion:**

There were no meaningful differences in PK between patients with normal renal function and ESRD in light of carfilzomib exposure–response relationships. These results continue to support dosing recommendation that no starting dose adjustment of carfilzomib appears warranted in patients with baseline renal impairment.

## Introduction

Renal insufficiency is a common and often severe complication in multiple myeloma [1, 2]. Several factors can contribute to renal insufficiency in multiple myeloma, including hypercalcemia and myeloma cast nephropathy. The most common, and in many cases irreversible, is immunoglobulin light chain damage to tubular cells [3, 4]. Two demographic studies evaluating a combined 2380 patients with newly diagnosed multiple myeloma found that nearly 50% of patients had impaired renal function as determined by estimated creatinine clearance (CrCL) at presentation, approximately 15% of whom had severe renal insufficiency with serum creatinine levels of >2.3 mg/dL [5, 6].

Patients with renal failure typically have more advanced disease, lower response rate, and decreased survival following chemotherapy versus normal renal function patients [1, 6]. The presence of renal impairment can limit treatment options or complicate dosing of drugs, leading in some cases to higher incidence or exacerbation of adverse events (AEs) [7].

Carfilzomib, an epoxyketone proteasome inhibitor, is approved in the United States (US), Canada, Australia, European Union (EU), and other jurisdictions for the treatment of relapsed or refractory multiple myeloma [8]. Studies on carfilzomib’s metabolism and elimination indicated that renal clearance is a minor component of overall plasma clearance of the parent compound [9, 10]. Following intravenous (IV) administration, carfilzomib is rapidly and extensively metabolized in patients with multiple myeloma or solid tumors [9]. Similar to the metabolic profile in rats and monkeys [10], the most abundant metabolites of carfilzomib in human plasma and urine collected from patients with hematological malignancies are peptide fragments of carfilzomib and carfilzomib diol, indicating that peptidase cleavage and epoxide hydrolysis are the principal pathways of metabolism [9]. Cytochrome P450 enzymes do not play a significant role in the overall metabolism of carfilzomib. In addition, the major metabolites of carfilzomib (M14, M15, and M16) lack an epoxyketone pharmacophore, and do not have activity as proteasome inhibitors [9, 10]. Evaluation of the pharmacokinetics (PK) of carfilzomib following IV administration at doses from 15 to 56 mg/m^2^ found that it was rapidly cleared from the systemic circulation, with a half-life of ≤1 h in patients [8, 9, 11]. Systemic clearance of 151 to 263 L/h has been described, exceeding hepatic blood flow and suggesting largely extrahepatic clearance. Within 24 h, approximately 25% of a dose of carfilzomib is excreted via urinary metabolites, with negligible (0.3% of the total dose) urinary and fecal excretion of the parent compound [8]. These studies suggest that renal impairment is expected to have minor impact on overall clearance of the parent compound.

The potential impact of renal impairment on PK of carfilzomib has been previously studied in patients with multiple myeloma at doses of 15 and 20 mg/m^2^ (IV infusion over 2–10 min) [11]. The results show no apparent differences in carfilzomib (15 or 20 mg/m^2^) clearance, area under the concentration time curve (AUC), and maximum plasma concentration (*C*_max_) between patients with normal versus those with mild, moderate, or severe renal impairment (classified by CrCl: >80 mL/min [normal], 50–80 mL/min [mild impairment], 30–49 mL/min [moderate impairment], and <30 mL/min [severe impairment]), including patients with end-stage renal disease (ESRD) on hemodialysis [11]. Recent data from the phase 3 ENDEAVOR study demonstrated that carfilzomib treatment at a dose of 56 mg/m^2^ as a 30-min IV infusion in multiple myeloma patients have shown promising clinical results, leading to approval of this dose regimen in the US, EU, and other jurisdictions [12]. The ENDEAVOR study enrolled subjects with varying degrees of renal impairment, as measured by CrCL, as low as 15 mL/min. Given that ESRD patients were excluded from the ENDEAVOR study, to support dose recommendation following the carfilzomib dose of 56 mg/m^2^ in patients with varying degrees of renal impairment, the current study was conducted to assess the influence of ESRD on the PK parameters of carfilzomib at doses of 27 and 56 mg/m^2^ in patients with relapsed multiple myeloma [12].

## Materials and methods

### Patients

Patients ≥18 years of age with relapsed multiple myeloma were eligible for enrollment into 1 of 2 groups: ESRD or normal renal function. Patients with clinically diagnosed ESRD must have been receiving hemodialysis for ≥1 month prior to enrollment and must have been stable (i.e., without acute complications). Patients with normal renal function had a calculated CrCL ≥75 mL/min at screening using the Cockcroft–Gault equation (normalization of the CrCL by body surface area was also ≥75 mL/min/1.73 m^2^ in all patients in the PK-evaluable cohort receiving the 56-mg/m^2^ dose). Patients must have had evaluable disease, with serum M-protein ≥0.5 g/dL or urine M-protein ≥200 mg/24 h; in patients without detectable serum or urine M-protein, serum free light chain (SFLC) >100 mg/L (involved light chain) and an abnormal kappa/lambda (*κ*/*λ*) ratio of <0.26 for patients with monoclonal *λ* free light chain (FLC) or >1.65 for patients with monoclonal *κ* FLC were required. Other inclusion criteria included having received ≥1 prior treatment regimen or line of therapy for multiple myeloma; Eastern Cooperative Oncology Group (ECOG) performance status 0–2; adequate hepatic function within 21 days prior to cycle 1 day 1 with bilirubin <1.5 times the upper limit of normal (ULN), and aspartate aminotransferase and alanine aminotransferase <3 times the ULN; absolute neutrophil count ≥1.0 × 10^9^/L, hemoglobin ≥8 g/dL, and platelet count ≥50 × 10^9^/L (or ≥30 × 10^9^/L if bone-marrow disease involvement was >50%) documented within 21 days prior to enrollment. Prior therapy with carfilzomib was allowed as long as the patient had at least a partial response (PR) to prior carfilzomib therapy, did not discontinue carfilzomib therapy due to toxicity, and had a ≥6-month treatment-free interval from last dose of carfilzomib received (patients in post-treatment follow-up of a clinical trial, however, were excluded). Prior therapy with a bortezomib-containing regimen was allowed and provided that the patient had a ≥21-day bortezomib treatment-free interval from last dose received until first study treatment.

Exclusion criteria included myocardial infarction within 6 months of enrollment, as well as current or history of congestive heart failure (New York Heart Association class III or IV), symptomatic ischemia, or uncontrolled conduction abnormalities. Patients were excluded if they had known human immunodeficiency virus or active hepatitis B or C virus infection; neuropathy of grade ≥2 severity; active infection requiring antibiotics; antiviral (except against hepatitis B) or antifungal treatment, or uncontrolled hypertension or diabetes within 2 weeks of enrollment; and any investigational or approved chemotherapy, or focal radiotherapy within 1 week, immunotherapy within 3 weeks, or major surgery within 3 weeks of enrollment.

### Study design

This was a phase I, multicenter (13 sites in US, Canada, and Australia), open-label, nonrandomized, comparative PK study of carfilzomib in patients with multiple myeloma and normal renal function or ESRD on hemodialysis (NCT01949532). The primary objective was to assess the influence of ESRD on AUC, from time 0 to the last concentration measured (AUC_0−last_) and from time 0 extrapolated to infinity (AUC_0−inf_) of carfilzomib 56 mg/m^2^ at cycle 2 day 1 in patients with relapsed multiple myeloma. Secondary objectives were to compare between patient cohorts additional PK parameters of carfilzomib at cycle 2 day 1, including *C*_max_, time to maximum concentration (*t*_max_), clearance, terminal half-life (*t*_1/2_), volume of distribution at steady state, and mean residence time; compare between patient cohorts PK parameters of carfilzomib 27 mg/m^2^ at cycle 1 day 16; evaluate PK parameters for carfilzomib’s major metabolites (PR-389/M14, PR-413/M15, and PR-519/M16) to help characterize metabolism and elimination profile of carfilzomib; and evaluate the safety and tolerability of carfilzomib. Exploratory objectives included evaluation of ORR (defined as PR or better) and clinical benefit rate [CBR; defined as ORR + minimal response (MR)], and estimation of duration of response (DOR) and PFS.

The protocol and informed consent document were reviewed and approved by each study center’s Institutional Review Board or Independent Ethics Committee. This study was conducted in accordance with International Conference on Harmonisation (ICH) Good Clinical Practice (GCP) regulations. All patients provided written informed consent prior to undergoing any protocol-specific screening procedures or treatments.

**Fig. 1:**
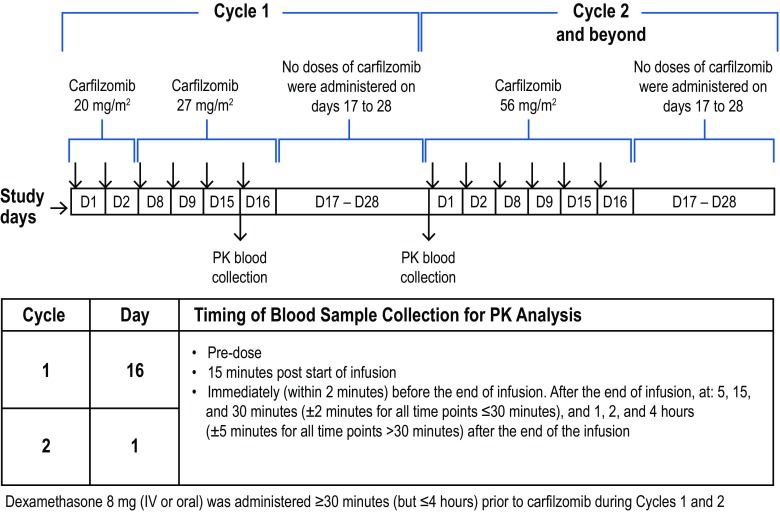
Treatment regimen and PK evaluation protocol. ^a^ *D* day, *IV* intravenous, *PK* pharmacokinetic

Patients received IV carfilzomib (as a 30-min infusion) in 28-day cycles (Fig. 1). All received carfilzomib 20 mg/m^2^ on days 1 and 2 of cycle 1 and escalated to 27 mg/m^2^ on day 8, 9, 15, and 16 of cycle 1. If tolerated, carfilzomib dose was increased to 56 mg/m^2^ starting on day 1 of cycle 2 and continued on the same schedule (day 1, 2, 8, 9, 15, and 16 of each cycle). Oral or IV dexamethasone 8 mg/day was given on the same days as carfilzomib during cycles 1 and 2 but was optional in cycles 3 and beyond. IV hydration was given immediately prior to and following carfilzomib dosing during cycle 1 and at the investigator’s discretion in cycle 2 and higher; hydration consisted of 250 to 500 mL IV normal saline or other appropriate IV fluid.

It was strongly recommended that valacyclovir (or an equivalent antiviral) and lansoprazole (or an equivalent proton pump inhibitor) be administered throughout the study in accordance with the manufacturer’s prescribing information or the investigator’s standard of care. Patients were permitted to continue carfilzomib until confirmed progressive disease (PD), unacceptable toxicity, withdrawal of consent, study closure, or death.

### Assessments

Blood samples were collected for PK assessments for carfilzomib and its major metabolites (metabolites PR-389/M14, PR-413/M15, and PR-519/M16) (Fig. 1). Samples were collected at the following timepoints on cycle 1 day 16 (27 mg/m^2^ dose) and cycle 2 day 1 (56 mg/m^2^ dose) or on subsequent infusion days when the intended dose levels were administered: before infusion; 15 min after start of infusion; within 2 min before the end of infusion; and at 5, 15, and 30 min (±2 min for all timepoints ≤30 min), and 1, 2, and 4 h (±5 min for all timepoints >30 min) after the end of the infusion. Safety was assessed throughout the study and included the monitoring of AEs, clinical laboratory evaluations, electrocardiograms, and vital signs. Each evaluable patient’s response to treatment or progression over the course of the study was evaluated by the investigator according to the International Myeloma Working Group, International Uniform Response Criteria for Multiple Myeloma [13, 14] and European Group for Blood and Marrow Transplantation [1, 6] at day 1 of each cycle.

### Statistical analysis

Descriptive statistics were provided for selected demographics, safety, and PK data, with all statistical summaries and analyses performed in SAS^Ⓡ^ version 9.1 or higher (SAS Institute Inc., Cary, NC). The primary PK analyses and other PK parameters were performed using the PK-evaluable population. Plasma PK parameters of carfilzomib and metabolites were computed in Phoenix WinNonlin^Ⓡ^ Enterprise version 5.2 or higher. To assess the effect of ESRD relative to normal renal function on PK parameters (AUC_0−last_ and AUC_0−inf_), an analysis of variance (ANOVA) of the ln-transformed plasma PK parameters was performed, with renal impairment as a fixed effect. Point estimates (geometric mean ratios) for the PK parameters were calculated by exponentiation of the differences in the least-squares means (LSM), using ln-transformed data, between the ESRD cohort (test) and normal renal function cohort (reference). The 90% confidence intervals (CIs) of the geometric mean ratios were transformed similarly by exponentiation of the corresponding 90% CI for the difference between the LSM calculated for the ln-transformed values.

Safety was assessed for patients who received ≥1 dose of carfilzomib. AEs were graded using the National Cancer Institute Common Terminology Criteria for Adverse Events version 4.03 and mapped to preferred term and system organ class using the Medical Dictionary for Regulatory Activities (MedDRA). ORR defined as the proportion of patients for whom the best overall response was stringent complete response, complete response, very good PR, or PR, along with the associated 95% exact binomial CI (Clopper–Pearson method) was determined. CBR, defined as the proportion of patients with the best overall response of MR or better, was likewise determined along with the 95% exact binomial CI.

## Results

### Patients and enrollment

The study was conducted between January 2014 and July 2015 (data cut-off, 12 October 2015). Of 36 screened patients, 26 (72%) were enrolled and treated: 15 with normal renal function and 11 with ESRD. Demographic and baseline characteristics were generally balanced between the cohorts (Table 1). Median patient age was 63 years (range 49–78 years) with 54% of patients <65 years of age. Most were Caucasian (81%), men (58%), and had an ECOG performance status of 1 (65%). Median time from initial diagnosis of multiple myeloma was 4.2 years, during which patients had received a mean of 3.5 prior regimens. Eighteen patients had received a prior transplant (87% of the normal renal function cohort and 45% of the ESRD cohort). Eleven patients with normal renal function (73%) and 4 patients with ESRD (36%) were refractory to the last prior systemic therapy. One patient (normal renal function cohort) received prior treatment with carfilzomib.

**Table 1:** Patient demographics and baseline disease characteristics

	Normal renal function (*n* = 15)	ESRD (*n* = 11)	Total (*N* = 26)
Sex, *n* (%)			
Male	10 (66.7)	5 (45.5)	15 (57.7)
Race, *n* (%)			
Asian	1 (6.7)	0	1 (3.8)
Black	1 (6.7)	1 (9.1)	2 (7.7)
White	12 (80.0)	9 (81.8)	21 (80.8)
Other/not reported	1 (6.7)	1 (9.1)	1 (3.8)
Age, years			
Median (range)	65 (53–77)	61 (49–78)	63 (49–78)
ECOG performance status, *n* (%)			
0	5 (33.3)	3 (27.3)	8 (30.8)
1	10 (66.7)	7 (63.6)	17 (65.4)
2	0	1 (9.1)	1 (3.8)
Time from initial diagnosis to informed consent, years			
Median (range)	4.6 (1.3–9.6)	4.0 (0.3–19.2)	4.2 (0.3–19.2)
Total number of prior regimens			
Median (range)	3 (1–9)	3 (1–7)	3 (1–9)
*n* (%)			
1	2 (13.3)	3 (27.3)	5 (19.2)
2	3 (20.0)	1 (9.1)	4 (15.4)
3	4 (26.7)	4 (36.4)	8 (30.8)
>3	6 (40.0)	3 (27.3)	9 (34.6)

*ECOG* Eastern Cooperative Oncology Group, *ESRD* end-stage renal disease, *Max* maximum, *Min* minimum

### PK carfilzomib

The PK-evaluable population overall included 23 patients (Table 2): 13 normal renal function and 10 ESRD patients. Three patients (2 with normal renal function and 1 ESRD patient) were excluded due to documented deviations from the protocol or PK laboratory manual related to PK sampling, namely, the carfilzomib infusion line was used for PK blood draws. One patient with normal renal function was included in the PK evaluation; however, this patient was excluded from the summary statistics, given that PK samples had been taken from the infusion arm (distal to the infusion site) on both cycle 1 day 16 and cycle 2 day 1, resulting in concentrations substantially higher than those in other patients.

**Table 2:** Carfilzomib PK parameters following IV administration of carfilzomib (PK-evaluable population)

PK parameters	27 mg/m^2^		56 mg/m^2^	
	Normal (*n* = 13)	ESRD (*n* = 9)	Normal (*n* = 10)	ESRD (*n* = 8)
AUC_0−last_ Geo mean, h·ng/mL (CV%)	344 (24.8)	480 (36.0)	563 (41.9)	747 (143.9)
Geo mean ratio (90% CI)	139.72 (112.41–173.66)		132.75 (70.60–249.63)	
AUC_0−inf_ Geo mean, h·ng/mL (CV%)	347 (26.3)^a^	479 (46.6)^b^	563 (41.8)	752 (144.7)
Geo mean ratio (90% CI)	138.09 (102.77–185.54)		133.62 (70.93–251.73)	
*C*_max_ Geo mean, ng/mL (CV%)	819 (29.8)	1022 (37.2)	1389 (26.8)	1567 (128.8)
*T*_max_ median, h (range)	0.58 (0.47–0.73)	0.47 (0.23–0.75)	0.47 (0.25–0.73)	0.47 (0.25–0.58)
*t*_1/2_ median, h (range)	0.39 (0.09–0.60)^a^	0.99 (0.92–16.0)^b^	0.34 (0.11–0.50)	1.25 (0.06–3.31)
CL Geo mean, L/h (CV%)	146 (23.0)	93.3 (56.8)	179 (38.9)	134 (136.9)
MRT Geo mean, h (CV%)	0.222 (16.6)^a^	0.426 (152.2)^b^	0.135 (62.6)	0.245 (79.9)
*V*_ss_ Geo mean, L (CV%)	32.0 (29.7)	53.0 (185.5)	24.1 (44.8)	32.8 (133.9)

*AUC*_*0−inf*_ area under the concentration time curve from time 0 extrapolated to infinity, *AUC*_*0−last*_ area under the concentration time curve from time 0 to last concentration measurement, *CI* confidence interval, *CL* clearance, *C*_*max*_ maximum plasma concentration, *CV* coefficient of variation, *ESRD* end-stage renal disease, *Geo mean* geometric mean, *IV* intravenous, *MRT* mean residence time, *PK* pharmacokinetic, *t*_*1/2*_ half-life, *t*_*max*_ time to maximum plasma concentration, *V*_*ss*_ volume of distribution at steady state

^a^ *n* = 11, ^b^ *n* = 6

**Fig. 2:**
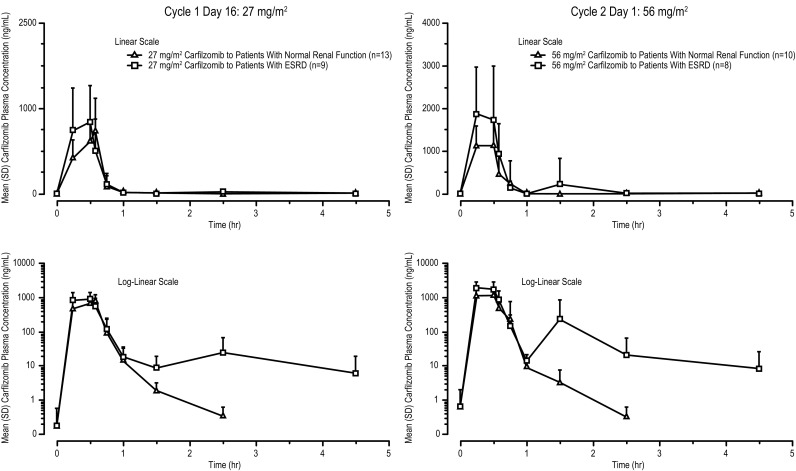
Mean (+SD) plasma concentration–time profiles of carfilzomib following IV administration of carfilzomib (linear and semi-log plots) (PK-evaluable population). *Error bars* represent standard deviation. *ESRD* end-stage renal disease, *IV* intravenous, *LOQ* limit of quantitation, *SD* standard deviation

Mean plasma concentration–time profiles of carfilzomib following IV infusion are shown in Fig. 2. Following IV administration, the plasma concentration of carfilzomib decreased rapidly in a biphasic manner. Peak concentrations of carfilzomib were observed most often around the end of the infusion; at approximately 0.5 h after start of infusion. Concentrations of carfilzomib then declined rapidly with a median *t*_1/2_ of approximately 0.4 and 0.3 h at 27 and 56 mg/m^2^, respectively, in the normal renal function cohort and 1.0 and 1.3 h, respectively, in the ESRD cohort. Mean values showed a dose-dependent increase in mean AUC_0−last_ and *C*_max_ of carfilzomib between 27 and 56 mg/m^2^ in both cohorts (Table 2). A trend for a higher AUC and *C*_max_ value for ESRD patients versus normal renal function patients at both carfilzomib doses were observed (Table 2). However, high interpatient variability was discerned in both groups (up to 100%) and exposure values in patients with ESRD were essentially within the range observed in patients with normal renal function. The effect of renal impairment (ESRD) on PK parameters of carfilzomib relative to normal patients was assessed using an ANOVA of the ln-transformed carfilzomib AUC_0−last_ and AUC_0−inf_ at doses of 27 and 56 mg/m^2^ (Table 2). Following IV administration of 27 mg/m^2^ carfilzomib, the estimated geometric mean ratio (%) for patients with ESRD compared with patients with normal renal function were 139.7% (90% CI 112.4–173.7) and 138.1% (90% CI 102.8–185.5) for AUC_0−last_ and AUC_0−inf_, respectively. Following an IV infusion of 56-mg/m^2^ carfilzomib on cycle 2 day 1, the estimated geometric mean ratio for patients with ESRD compared with patients with normal renal function was 132.8% (90% CI 70.6–249.6) and 133.6% (90% CI 70.9–251.7) for AUC_0−last_ and AUC_0−inf_, respectively.

### PK of major metabolites

For each metabolite, dose-dependent increases in mean AUC_0−last_ and *C*_max_ were observed. At a given dose level, the increases were greater in ESRD versus normal renal function for PR-389/M14 and PR-413/M15 (by ~fourfold and <twofold, respectively), but were comparable between the two groups for PR-519/M16.

### Efficacy

The efficacy-evaluable population consisted of 14 patients with normal renal function and 10 patients with ESRD who achieved a response of at least PR or MR. The ORR for the entire study was 50% (95% CI 29.1–70.9) with 60% (95% CI 26.2–87.8) for the ESRD cohort (n = 6 patients achieving PR or better) and 43% (95% CI 17.7–71.1) for the normal renal function cohort (n = 6 patients achieving PR or better) (Table 3). Overall CBR rate was 54%, as 1 additional patient with normal renal function achieved an MR. DOR ranged from 0.9 (censored) to 13 months. Ten patients had a PFS of ≥6 months (6 of 14 with normal renal function and 4 of 10 patients with ESRD), including 4 patients with a PFS >12 months (*n* = 3 and *n* = 1, respectively). As of the data cutoff (12 October 2015), 6 patients remained on treatment.

**Table 3:** Best overall response as determined by investigator (response-evaluable population)

	Normal renal function (*n* = 14)	ESRD (*n* = 10)	Total (*n* = 24)
Best overall response, *n* (%)			
Stringent complete response	0 (0.0)	0 (0.0)	0 (0.0)
Complete response	0 (0.0)	0 (0.0)	0 (0.0)
Very good partial response	3 (21.4)	1 (10.0)	4 (16.7)
Partial response	3 (21.4)	5 (50.0)	8 (33.3)
Minimal response	1 (7.1)	0 (0.0)	1 (4.2)
Stable disease	3 (21.4)	2 (20.0)	5 (20.8)
Progressive disease	2 (14.3)	2 (20.0)	4 (16.7)
Not evaluable	2 (14.3)	0 (0.0)	2 (8.3)
Overall response rate^a^ (95% CI)	42.9 (17.7–71.1)	60.0 (26.2–87.8)	50.0 (29.1–70.9)
Clinical benefit rate^b^ (95% CI)	50.0 (23.0–77.0)	60.0 (26.2–87.8)	54.2 (32.8–74.4)

Disease response and progression were determined using the International Myeloma Working Group Uniform Response Criteria, except for minimal response, which was based on the European Group for Blood and Marrow Transplantation criteria

*CI* confidence interval, *ESRD* end-stage renal disease

^a^Partial response or better

^b^Minimal response or better

### Safety

All 26 dosed patients were included in the safety evaluation population. At data cutoff, median exposure was 14.1 weeks (4.0 cycles) and 12.1 weeks (3.0 cycles) for patients with normal renal function versus ESRD, respectively.

All patients reported at least one AE during treatment. Treatment-related AEs were reported for 80% (12/15) of patients with normal renal function and 73% (8/11) of patients with ESRD (Table 4). Treatment-related grade ≥ 3 AEs were reported for 47% (7/15) and 55% (6/11) of normal renal function and ESRD patients, respectively, with thrombocytopenia being the most common (>3 patients) grade ≥3 treatment-related AE (26.7% in normal renal function patients and 9.1% in ESRD patients).

**Table 4:** Adverse event summary (safety population)

AE^a^, *n* (%)	Normal renal function (*n* = 15)	ESRD (*n* = 11)	Total (*N* = 26)
Any AE^b^	15 (100)	11 (100.0)	26 (100.0)
Any grade ≥ 3 AE	12 (80.0)	9 (81.8)	21 (80.8)
Any grade ≥3 AE reported in >3 patients			
Thrombocytopenia	7 (46.7)	2 (18.2)	9 (34.6)
Anemia	4 (26.7)	5 (45.5)	9 (34.6)
Pneumonia	2 (13.3)	2 (18.2)	4 (15.4)
Treatment-related AE^b^	12 (80.0)	8 (72.7)	20 (76.9)
Treatment-related AE reported in >3 patients			
Fatigue	3 (20.0)	5 (45.5)	8 (30.8)
Nausea	4 (26.7)	2 (18.2)	6 (23.1)
Dyspnea	4 (26.7)	1 (9.1)	5 (19.2)
Thrombocytopenia	4 (26.7)	1 (9.1)	5 (19.2)
Diarrhea	3 (20.0)	1 (9.1)	4 (15.4)
Treatment-related grade ≥3 AE	7 (46.7)	6 (54.5)	13 (50.0)
Treatment-related grade ≥3 AE reported in >3 patients			
Thrombocytopenia	4 (26.7)	1 (9.1)	5 (19.2)
Treatment-related serious AE	5 (33.3)	2 (18.2)	7 (26.9)

*ESRD* end-stage renal disease

^a^Adverse events (AEs) are treatment-emergent AEs, defined as any AE with an onset date between the date of first dose and 30 days after the date of last dose of carfilzomib, ^b^Any-grade AE reported in ≥30% of patients, ^c^Treatment-related AEs are treatment-emergent AEs considered related to carfilzomib by the investigator, including those with unknown relationships

Twenty patients had discontinued treatment as of the data cutoff. The primary reasons for treatment discontinuation were disease progression (20 and 36% in patients with normal renal function and ESRD, respectively) and AEs (40 and 0%, respectively). Six patients with normal renal function discontinued the study due to AEs, compared with none of the ESRD patients. AEs resulting in treatment discontinuation in >1 patient included general health deterioration (*n* = 2; not considered related to treatment). Four of the normal renal function patients experienced AEs that were considered related to treatment and led to study discontinuation: acute kidney injury, acute interstitial pneumonitis, and thrombocytopenia (all in a single patient); hypoxia; pulmonary hypertension; and microangiopathic hemolytic anemia. Five patients died during the study (3 normal renal functions; 2 ESRD); however, none were considered related to carfilzomib treatment. One patient with normal renal function died as a result of cardiorespiratory arrest, with underlying disease cited as a possible explanation by the investigator. The other 4 deaths (*n* = 2 per cohort) were due to disease progression.

## Discussion

In this phase I trial, trends toward higher exposure (AUC and *C*_max_), longer *t*_1/2_, and slower clearance for carfilzomib 27- and 56-mg/m^2^ doses were observed in patients with ESRD relative to those with normal renal function without an apparent increase in AEs. Following IV administration of carfilzomib at doses of 27 and 56 mg/m^2^, mean carfilzomib systemic exposures (AUC_0−last_ and AUC_0−inf_) were approximately 33% higher, but not statistically different in patients with ESRD compared with normal renal function patients. High interpatient variability was discerned (up to 100%) and exposure values in patients with ESRD were essentially within the range observed in patients with normal renal function.

The results of the current study are consistent with those in a prior renal impairment study evaluating single- and repeat-dose administration of carfilzomib 15 and 20 mg/m^2^ in relapsed multiple myeloma patients, which found no marked differences in *C*_max_ and AUC between normal renal function patients and those with mild, moderate, or severe renal impairment or ESRD [11]. PK variability was also found to be high with overlapping exposures observed in patients with normal renal function and those with varying degrees of renal impairment; a similar trend of increasing exposure in patients with ESRD was also observed. The high PK variability seen in some carfilzomib clinical trials is likely due in large part to the challenge of PK collection logistics for a short half-life molecule. Carfilzomib has a mean terminal *t*_1/2_ between 0.4 and 1.2 h and has a biphasic PK profile with a rapid concentration decline (two orders of magnitude) in the first 15 min following the end of the infusion. In particular, a slight difference in the timing of PK collection near the end of infusion (to characterize the peak concentration) can result in large concentration differences and high variability in PK parameters.

Our findings are also consistent with those of a population PK model based on carfilzomib PK data across 9 clinical trials (*n* = 203 normal renal function; *n* = 240 mild impairment; *n* = 144 moderate impairment; *n* = 24 severe impairment; *n* = 16 ESRD) [15]. CrCL and degree of renal impairment (mild, moderate, and severe) were among the 11 covariates applied to the model. Renal impairment had a minimal impact on PK parameters for both carfilzomib doses of 27 and 56 mg/m^2^. The pharmacokinetic results of both renal impairment studies are consistent with the clearance mechanism of carfilzomib; carfilzomib is primarily eliminated by metabolism to M14 before urinary excretion, and renal clearance is a minor component of the overall plasma clearance of the parent compound. In addition, an exposure–response analysis including patients from 5 phase Ib/II and 2 phase III studies (*N* = 576) showed that after adjusting for baseline characteristics and prognostic factors, higher exposure (cycle 1 average AUC) of carfilzomib was associated with improved ORR/CBR across a dose range of 15 to 20/56 mg/m^2^, and increasing exposures are not associated with increasing risk of adverse events [15]. Thus, a trend toward an increased AUC (a mean increase of 33%) in patients with ESRD is unlikely to be clinically relevant in light of intrinsic pharmacokinetic variability and exposure–response relationship shown for carfilzomib [15].

Evaluation of the major metabolites of carfilzomib found that systemic exposure to PR-519/M16 was similar between the ESRD and normal renal function cohorts; however, exposure to PR-413/M15 and PR-389/M14 appeared to be elevated in patients with ESRD. It is important to note that the M14 and M15 metabolites, similar to M16, lack biologic proteasome inhibition activity [9, 10]; thus, these metabolites do not contribute to the pharmacology activity observed in the patients.

Safety findings were generally similar between patients with and without ESRD, and the observed AE profile was generally consistent with the known safety profile of carfilzomib in the treatment of patients with relapsed multiple myeloma [11, 16, 17]. No ESRD patients withdrew from the study due to AEs. The incidence of acute renal failure has been reported more frequently in advanced relapsed and refractory multiple myeloma patients with lower baseline CrCL relative to those with higher baseline CrCL when receiving carfilzomib monotherapy [8] or carfilzomib combined with dexamethasone in the ENDEAVOR trial [12]. Overall, there are limited efficacy and safety data on patients with baseline CrCL <30 mL/min. Renal function should be monitored with regular measurements of serum creatinine and/or estimated CrCL.

The 50% ORR in the overall population was similar to those previously reported in trials of carfilzomib 56 mg/m^2^ infused for 30 min with or without dexamethasone for relapsed multiple myeloma (50% in a phase I trial and 55% in a phase II trial) [18, 19]. Together, the results of this study support current recommendations for carfilzomib that no starting dose adjustment is warranted for patients with baseline renal impairment including those with ESRD [8, 20].

In summary, relative to patients with normal renal function, ESRD patients on hemodialysis showed approximately 33% higher carfilzomib AUC following administration of carfilzomib at doses of 27 and 56 mg/m^2^. However, high interpatient variability was discerned and exposure values in patients with ESRD were essentially within the range observed in patients with normal renal function. Thus, no meaningful PK differences were observed between patients with normal renal function and patients with ESRD in light of intrinsic PK variability and exposure–response relationships of carfilzomib. The observed AE profile in this study was qualitatively consistent with the known safety profile of carfilzomib in the treatment of patients with relapsed multiple myeloma. In addition, the clinical benefit of carfilzomib was similar to other carfilzomib monotherapy studies and did not differ between patients with normal renal function and ESRD. Similar to the results of the previous renal impairment study at doses of 15 and 20 mg/m^2^, the results of the current study continue to support the label recommendation that no starting dose adjustment is warranted in patients with baseline renal impairment, including those with ESRD.
